# Neuromorphic Reservoir Computing with Memristive Nanofluidic Diodes

**DOI:** 10.1021/acs.nanolett.5c00853

**Published:** 2025-06-09

**Authors:** Sergio Portillo, Patricio Ramirez, Salvador Mafe, Javier Cervera

**Affiliations:** † Departamento de Física de la Terra i Termodinàmica, Universitat de València, E-46100 Burjassot, Spain; ‡ Departamento de Física Aplicada, 16774 Universitat Politécnica de València, E-46022 València, Spain; § Allen Discovery Center, Tufts University, Medford, Massachusetts 02155-4243, United States

**Keywords:** reservoir computing, neuromorphic, memristor, nanofluidics, nanopores

## Abstract

Memristive systems show conductance states modulated by past electrical stimuli acting as artificial synapses. Most neuromorphic computing systems are based on solid-state memristive devices that use physical environments and electrical carriers different from the ionic solutions characteristic of biochemical and bioengineering applications. Here, we use membranes with multiple nanopores showing different conductance states in an aqueous electrolyte as a model for reservoir computing (RC). To this end, the different membrane conductances obtained with distinct sequences of voltage pulses in the millisecond range are used for the identification of 10-digit *inputs* in the case of both correct and corrupted *inputs*. Using the current rectification of the nanofluidic conical diodes, we explore two additional options: (i) the use of the current and its sign instead of the conductance in the digit identification and (ii) the use of an antiparallel arrangement of two membranes instead of the single-membrane unit.

## Graphical abstract

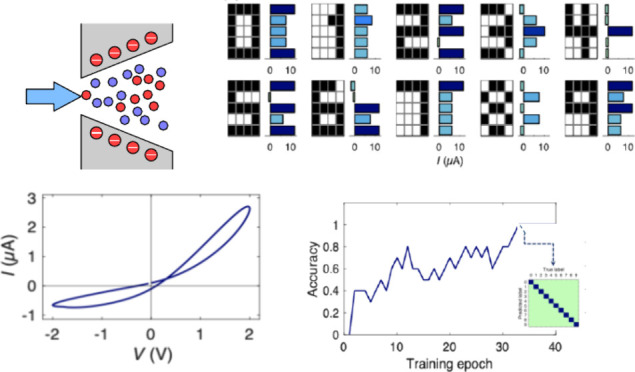

Memristor devices are characterized by conductance states that are modulated by past electrical stimuli such as voltage pulses, acting as artificial synapses potentially useful for neuromorphic computing.^1–5^ In particular, short-term memory effects can be useful for processing temporal input signals in reservoir computing (RC) networks,^6–13^ where learning is facilitated by the system training at the read-out stage. Although memristors can be fabricated on organic, inorganic, and hybrid materials, most implementations use solid-state devices based on metal oxide materials, transition-metal sulfides, ferroelectric materials, perovskites, etc.,^1–13^ that involve mechanisms such as electronic conduction, thermionic emission, vacancy migration, etc.^2–7^ Although organic polymer and biopolymer materials,^14^ including memristive biomolecular membranes that emulate key synaptic functions in RC,^15–17^ have also been used, most artificial synapses rely on physical environments and electrical carriers that differ from those of ionic aqueous solutions.^1–13^ This fact can be a shortcoming in biochemical and bioengineering applications characterized by different conduction mechanisms and comparatively slow time scales.^15–28^

Recently, micro- and nanofluidic iontronic systems that operate in aqueous electrolytes have been presented. For instance, the insertion of alamethicin peptides into a lipid bilayer allows conductive pathways that allow synapse-like dynamics^15^ and voltage-controlled memcapacitors^17^ where the bilayer can mimic the functionality of biomembranes. Also, conducting networks of fluidic nanochannels with remarkable RC properties have been demonstrated.^22^ In general, ionic systems show useful features such as mechanical and electrochemically regulated plasticity, spiking phenomena, and logic functionality.^19–35^ Here, we attempt to go a step further by considering a set of different nanopores under distinct voltages, current signs, and arrangements. The basic requirements for the nanofluidic conical pores used are rather general and can be readily met with current technology.^22,32–37^ Also, a broad range of steady-state and time-dependent electrical responses can be elicited by the available geometries and functionalizations of the pore surface.^29,33,34,38–40^

As a test, we consider the case of four-pulse train *inputs* (2 ms) that generate 2^4^ = 16 states. By using general RC procedures,^6,7,9,10,12,22^ these states allow one to identify a set of 10 digits characterized by 5 × 4 binary pixel images. In this arrangement implemented through a physical reservoir, only a one-layer neural network (the read-out layer) needs to be trained for a classification task. Thus, the training software is rather simple, and only a moderate number of training epochs are needed to achieve classification accuracies close to 100%. Corrupted noisy images can be recognized without further training. Also, we consider the effects of membrane variability on the RC performance.

Due to the current rectification of the nanofluidic conical diodes, we can explore also the reservoir performance when (i) the current and its sign is used instead of the conductance and (ii) the single membrane is replaced by an antiparallel arrangement of two membranes. Because the surface of the nanofluidic conical diodes can be functionalized with specific ligands that control the pore conductance,^14,34,36,37^ potential applications in signal diagnosis by parallel nanopore arrays should also be possible.

The multipore membranes with conical nanopores were fabricated by irradiation of a 12.5-μm-thick polyimide foil with single swift heavy ions.^32,33^ Subsequently, asymmetric track-etching techniques allowed to functionalize the pores with carboxylic moieties, which provided negative surface charges at neutral pH. Typical tip and base radii of the conical pores were on the order of 10 and 100 nm, respectively.^33^ The current (*I*)–voltage (*V*) curves of the membrane were measured with a couple of Ag|AgCl electrodes connected to a potentiostat (BioLogic SP-200). In order to eliminate electrical and mechanical perturbations, the electrochemical cell was confined to a double-shielded Faraday cage (Amuneal Manufacturing, Philadelphia, PA) and placed on an antivibration table (Technical Manufacturing Corporation, Peabody, MA). For the sake of reproducibility and operation, different multipore membrane samples with 1 cm^2^ exposed area were used. Also, single membrane and two antiparallel membrane arrangements were considered.

The ionic accumulation (voltage *V*_p_ > 0) and depletion (voltage *V*_n_ < 0) at the tip of the nanofluidic conical pores allow one to modulate the membrane conductance (Figure 1).^41^ The short-term plasticity of the conductance permits mapping of the different temporal *input* patterns into distinct reservoir states that can then be processed through a standard read-out function, as shown in Figure 1. Here, we use a typical RC arrangement,^6,7,9,10,12,22^ where the membranes are initially assumed to be identical. The final conductance states are then used to associate the different *inputs* to the distinct digit *outputs* by a read-out function that is trained by using the gradient descent method.

**Figure 1:**
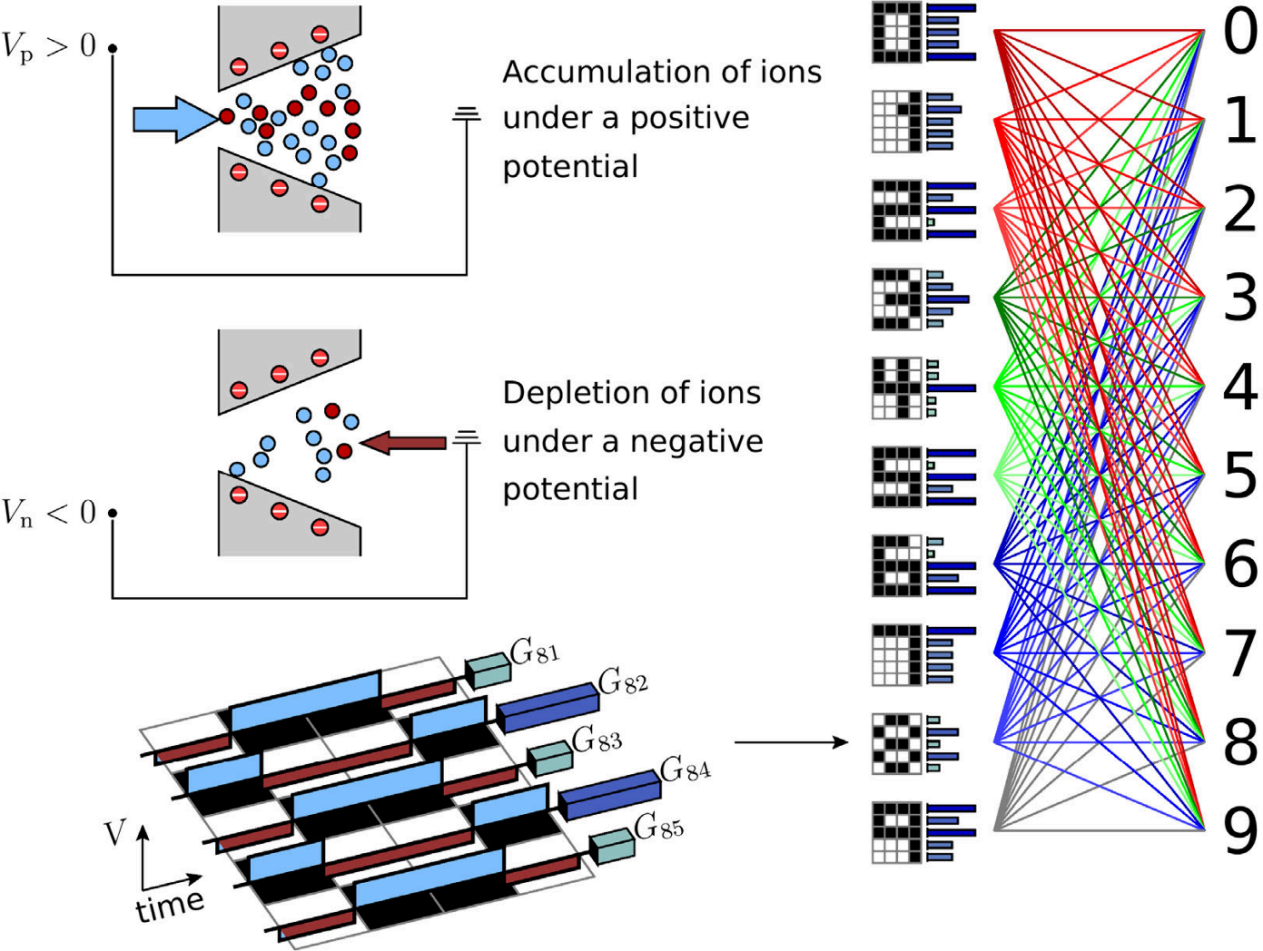
Mapping of the positive and negative voltage pulses (*input*) into the distinct reservoir states (*read-out*) permits to identify the patterns of 10 digits (*output*). The ionic accumulation (voltage *V*_p_ > 0, high membrane conductance) and depletion (voltage *V*_n_ < 0, low membrane conductance) in the nanofluidic conical pores provide the short-term plasticity of the membrane conductance needed for RC. The final conductance states corresponding to the digits *i* = 0, 1, ..., 9 and the assumed identical membranes *j* = 1, 2, ..., 5 are grouped in the conductance matrix *G*_*ij*_. These reservoir states are then processed by the read-out layer, which associates the different pulse *inputs* patterns to the distinct digit *outputs*, allowing an optimum separation between them.

The density of pores in the membrane is an important experimental parameter (Figure S1). Here, we have used samples with ca. 300 pores/cm^2^ and an exposed membrane area of 1 cm^2^. These samples show *I*–*V* curves with compensated capacitive (voltage *V* < 0) and inductive (*V* > 0) loops (Figure S2). When the number of pores decreases at a constant membrane area, the capacitive effects increase and tend to obscure the inductive effects. In this case, the conductance potentiation becomes too low to permit the implementation of RC with the nanopore network.

A set of different voltage pulses can change the final conductance state of the pores because the ions are pumped into or extracted from the pore solution following each pulse (Figure 1). The resulting ion accumulation or depletion depends on the sign of the voltage pulse. Also, the time needed for the pore solution to relax to the original state can be modulated by the amplitude and duration of the pulses in the sequence (Figure S3).^24,31^

To achieve the required accuracy in prescribed time periods, the RC system needs appropriate *output* separations for the *input* series, together with an efficient read-out function. Figure 2 displays the different final conductances of each individual membrane in every row sequence of pulses of 10-digit identification. The membrane is located in a 100 mM KCl solution, and the sequence of voltages includes 2 ms pulses of amplitudes *V*_p_ = 4 V and *V*_n_ = −2 V. Figure 2 also shows the RC read-out layer training procedure using the *G*_*ij*_ values as reservoir states. The confusion matrices for three different training epochs, together with the classification accuracy per epoch, are shown.

**Figure 2:**
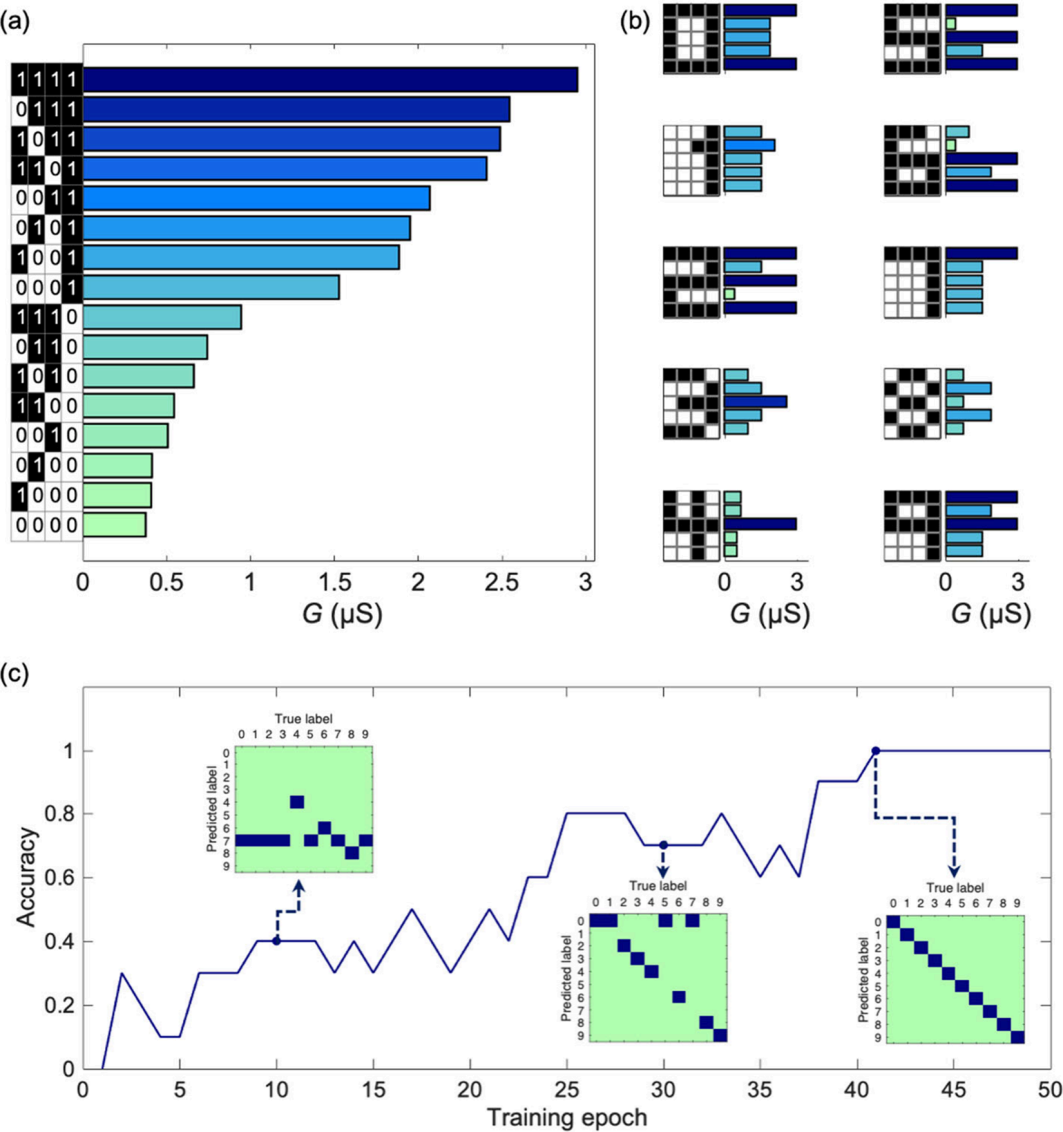
(a) Membrane final conductance values for the 2^4^ = 16 voltage pulse sequences. (b) Signature *G*_*ij*_ patterns for the binary 5 × 4 images of the digits from “0” to “9”. (c) Classification accuracy per training epoch and confusion matrices after 10, 30, and 41 training epochs. The predicted label for each digit is represented by a dark-blue square. The training of the read-out layer is based on the gradient descent method for the digit classification task.

Figure 3 considers the case of *input corrupted* digits and the corresponding confusion matrices. The conductance signatures for these corrupted patterns are simulated using a previously discussed phenomenological model.^27^ Here, the noisy images and the conductance patterns for two digits, whose images have one and two mismatched bits, respectively, are shown. These digits are not classified correctly in the confusion matrix. In Figure 3a, the noisy digit “5” is misclassified as “6”. In Figure 3b, the noisy digit “9” is misclassified as “5”. Parts c and d of Figure 3 show the average confusion matrix obtained with 1000 sets of noisy images. For one mismatched bit (Figure 3c), an average accuracy of 0.76 is achieved. For two mismatched bits, the identification worsens, giving an average accuracy of 0.59 (Figure 3d). However, the confusion matrix still preserves a clear diagonal character. Note that the classification has been completed with the same read-out layer obtained from the training procedure of Figure 2 with no further training.

**Figure 3:**
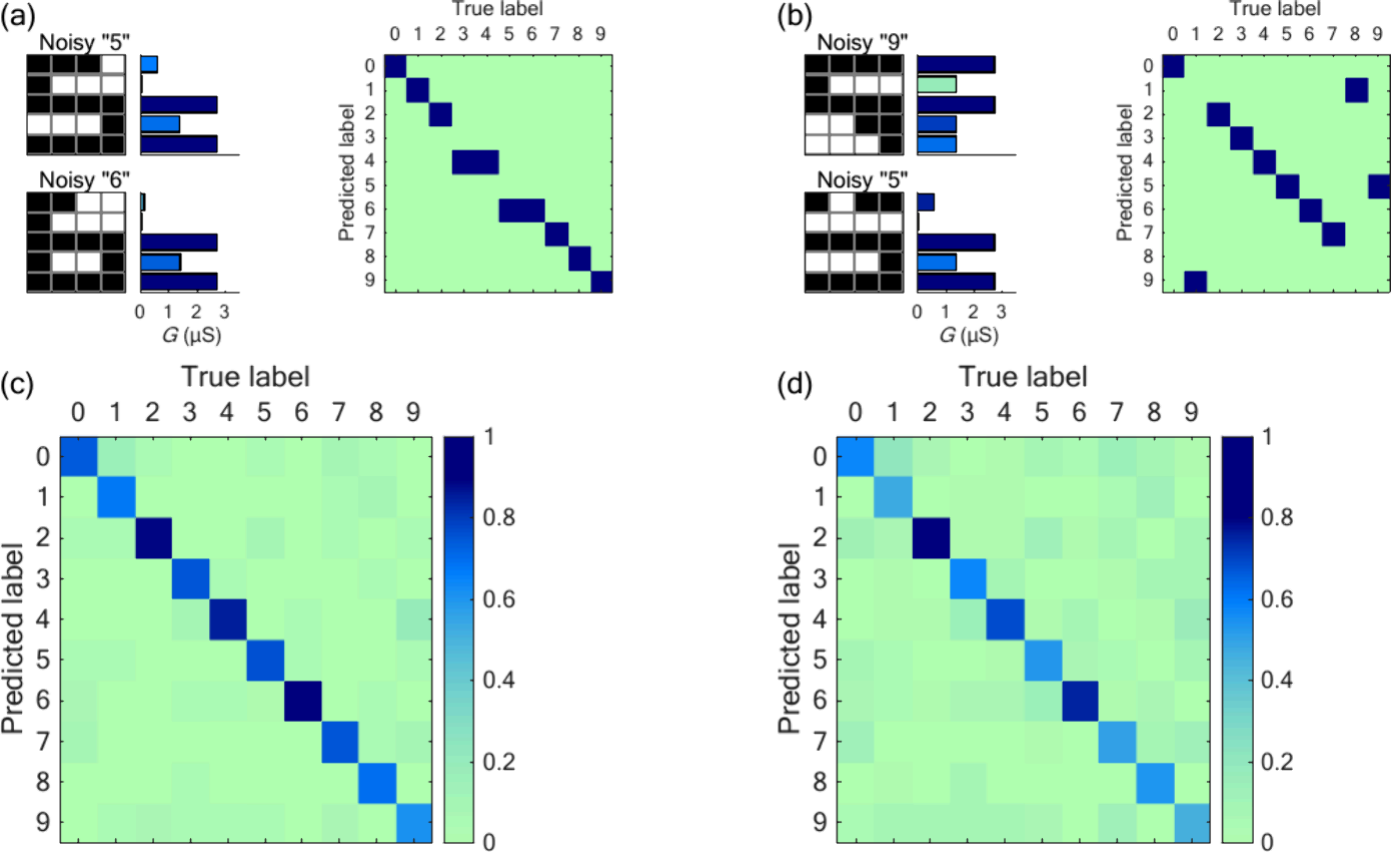
(a) Two patterns of a particular set of 1-bit corrupted digits and the confusion matrix. (b) Case of a particular set of 2-bit corrupted digits. (c) Average confusion matrix obtained over 1000 sets for the 1-bit corrupted case. (d) Case of 2-bit corrupted digits. While the digit identification worsens when the number of corrupted bits increases, the confusion matrix still shows significant average accuracies.

Figure 4 considers the effect of membrane variability on the RC performance. At the nanoscale, nominally identical nanopores showing exactly the same memristive properties are difficult to obtain. In addition, the nonlinear transformation of the physical response could not probably explore a large region of the RC state space in the case of almost identical pores. Thus, the data set should be extended by adding variability while keeping limited the number of laboratory realizations. In principle, we may use a large set of different memristors during long time runs, thus exploiting the inherently random variability of memristor responses. However, this procedure would demand extensive material and time-consuming procedures. As an alternative, we can use a virtual system that uses the experimental conductances obtained with the voltage pulses and the relaxation times as inputs^31^ (Figures S4 and S5). Here, the membrane variability is simulated by introducing random fluctuations in the experimental conductance values within a fixed range of variability σ_max_. In this way, we can simulate the implementation of five individually different membranes in the RC network. Finally, the effect of membrane variability on the RC performance is shown in Figure 4. Taken together, the results of Figures 2–4 clearly suggest that the hybrid procedure combining experimental and theoretical layers gives approximately valid results.

**Figure 4:**
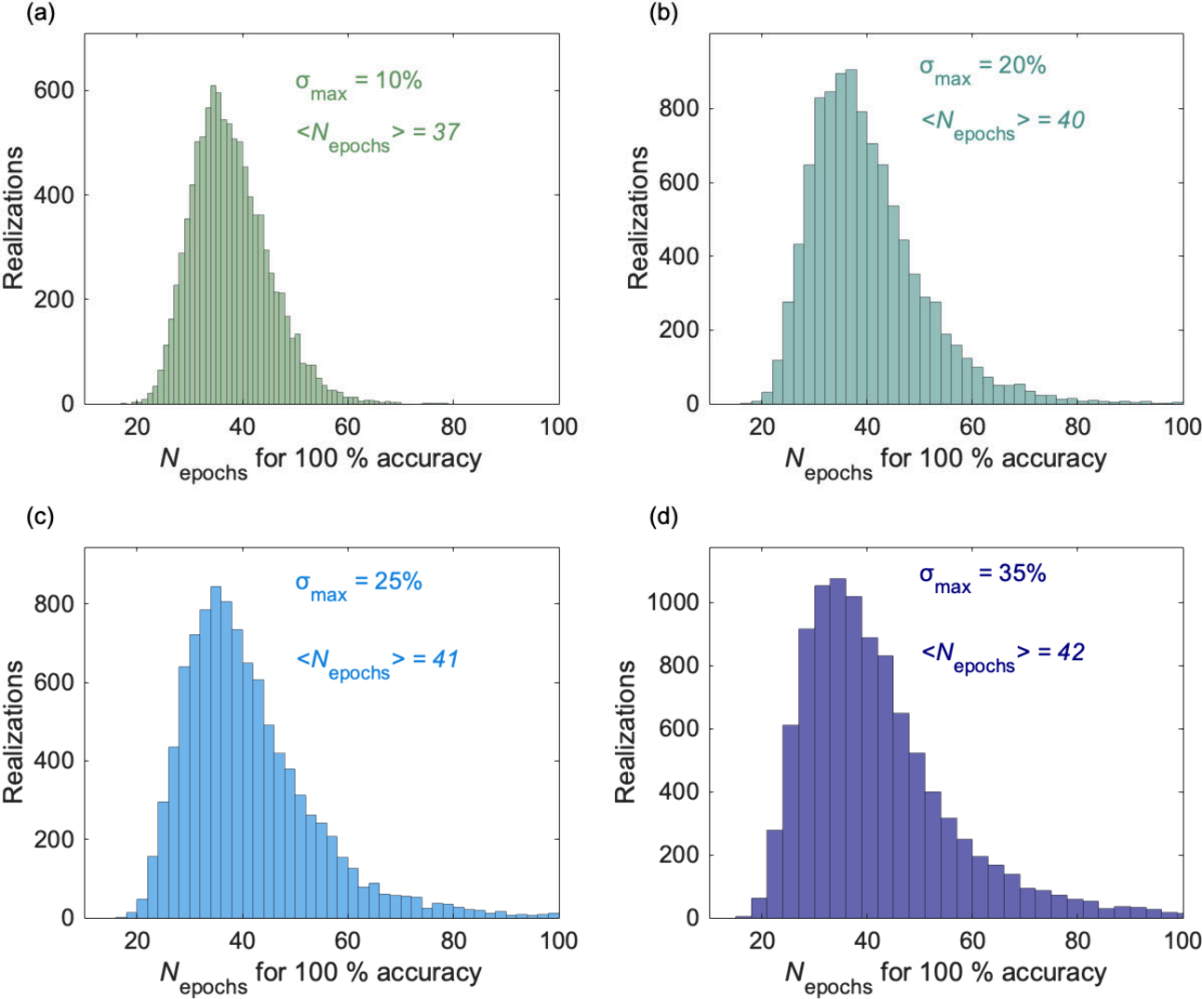
(a) Number of training epochs needed for a 100% classification accuracy over 10000 different realizations assuming a 10% maximum variability in the membrane conductance. (b) 20% maximum variability. (c) 25% maximum variability. (d) 35% maximum variability. The mean value of the needed epochs, ⟨*N*_epochs_⟩, for each distribution is shown.

We follow the gradient descent training procedure of Figure 2 to obtain the number of epochs needed to achieve a 100% classification accuracy for membrane variability in the range 10–35% (Figure 4). Here, 10000 different realizations, characterized by distinct values of the membrane conductances in Figure 2, are considered for σ_max_ values of 10% (Figure 4a), 20% (Figure 4b), 25% (Figure 4c), and 35% (Figure 4d). The mean value ⟨*N*_epochs_⟩ of the number of epochs needed for maximum accuracy is shown. Clearly, the RC performance does not need significantly higher training epochs than in the above cases, in agreement with previous simulations with different nanostructures.^42,43^ A moderate membrane variability in the range of 10–15% can even improve the system performance without compromising the training procedure (Figure 4).

Figure 5 shows a significant reduction in the number of training epochs when (i) the current and its sign is used, instead of the conductance, to define the reservoir states (Figure 5a–c) and (ii) the single membrane used for each of the five rows in the digit identification of Figure 1 is replaced by an antiparallel arrangement of two identical membranes (Figure 5d–f). In the case of a single membrane, the improvement obtained is limited by the small currents of negative voltages. However, this improvement is further increased by using the antiparallel configuration as the physical reservoir.

**Figure 5:**
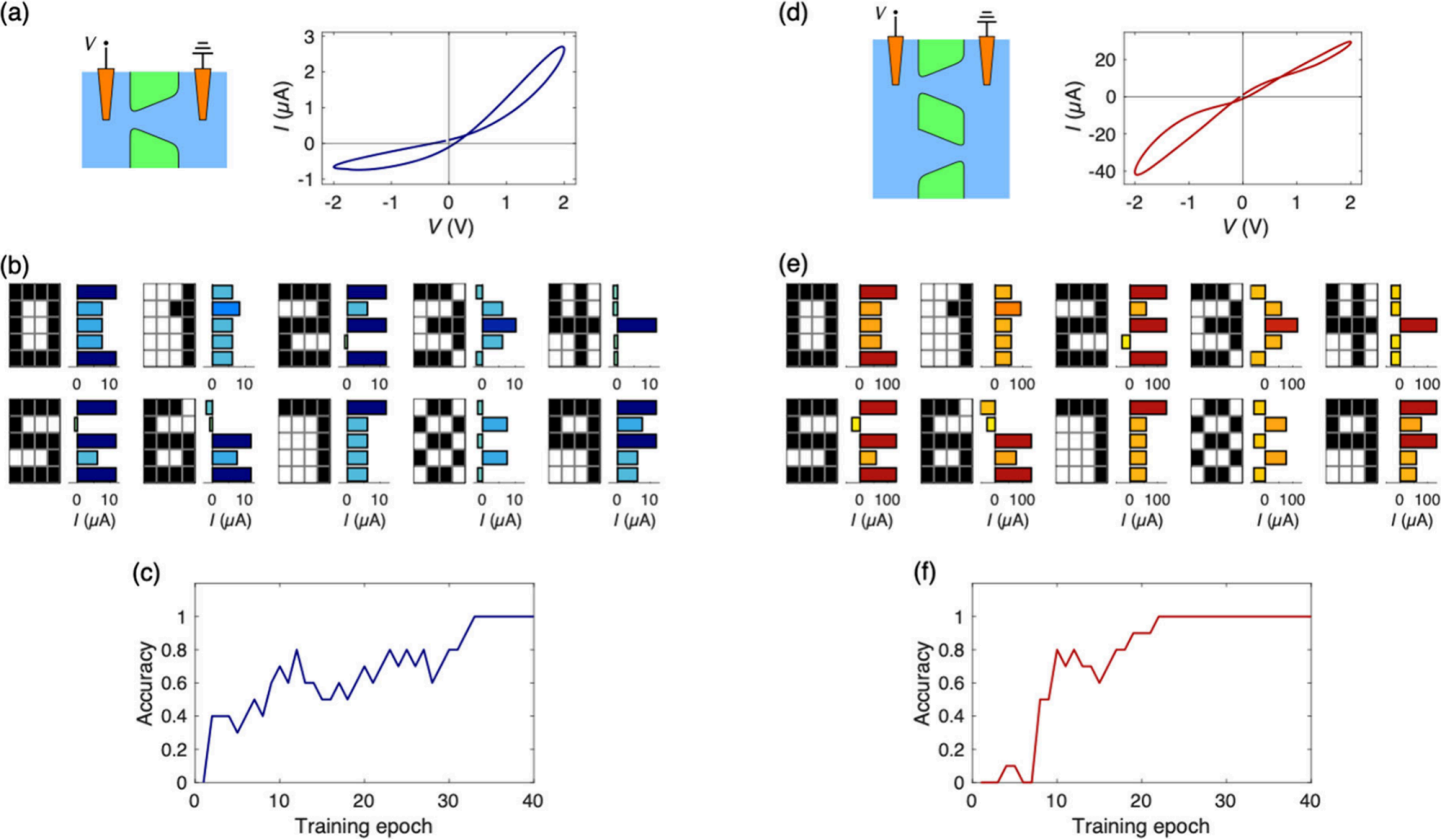
(a) Scheme of an individual conical pore in the membrane, the electrodes, and the experimental memristive (*I*)–voltage (*V*) curve obtained with a sinusoidal voltage wave of frequency 20 Hz. (b) Patterns of final currents obtained for each digit identification. A multipore membrane is considered for each of the five rows in the digit identification of Figure 1. (c) Accuracy vs training epoch using the current instead of the membrane conductance. (d) Case of an antiparallel arrangement of two identical membranes instead of only one membrane for each of the five rows forming each digit. The scheme of two individual conical pores, one in each membrane, the electrodes, and the experimental steady-state *I*–*V* memristive curve are shown. (e) Final currents obtained for each digit identification. (f) Accuracy vs training epoch using the current instead of the membrane conductance.

In a different but related field, artificial nanopores can mimic some of the ionic selectivity and current rectification functionalities of the ion channel proteins in biological membranes.^44^ Remarkably, the concerted action of two generic families of outward- and inward-rectifying voltage-gated channels also allows control of the dynamic balance between the respective outward and inward currents (Figure 5d,e), which regulates the cell response to electrical *inputs*.^44^

Note finally that the nanopore system used here is characterized by well-established operation modes, excellent stability and robustness, and a convenient interconnectivity of the individual components that allow one to implement a broad set of logical functions on the basis of the memristive conductance potentiation.^31^ The membrane response can be modulated not only by the frequency and amplitude of the driving signal but also by the external solutions (ionic concentration and pH values),^29,30^ which is reminiscent of physiological processes in cell membranes.^44^ These facts should facilitate signal conversion and information processing in bioengineering applications.

In summary, membranes with multiple nanopores in an aqueous electrolyte have been employed for neuromorphic RC. The different membrane conductances obtained with distinct sequences of voltage pulses are used for the identification of 10-digit *inputs*. The effects of corrupted *inputs* and membrane variability on the RC performance are also studied. Because of the current rectification of the nanofluidic conical diodes, significant improvement is obtained when (i) the current and its sign are used instead of the conductance and (ii) the single membrane in the digit identification is replaced by an antiparallel arrangement of two identical membranes. The use of conventional electrochemical cells with typical chemical and electrical signals, together with the fact that the surface of the nanofluidic conical diodes can be functionalized with specific ligands that regulate the pore conductance, suggests also potential applications in signal diagnosis by parallel nanopore arrays.

## Supplementary Material



## Data Availability

Data will be made available on reasonable request.

## Notes

Additional experiments concerning the effect of the number of pores, the memristive inductive and capacitative membrane responses, and the retention times, together with model theoretical results (PDF)
