# Supplementary material for: Neuromorphic Reservoir Computing with Memristive Nanofluidic Diodes
Source: Nano Lett. 2025 Jun 9;25(25):9928–34. doi: 10.1021/acs.nanolett.5c00853 (PMC12203634; doi:10.1021/acs.nanolett.5c00853)
Supplement: Supplementary file 1 [file nl5c00853_si_001.pdf]

Supporting Information:

Neuromorphic Reservoir Computing with Memristive Nanofluidic Diodes

Sergio Portillo,[a] Patricio Ramirez,[b,*] Salvador Mafe,[a,c] Javier Cervera[a,**]

[a]Dept. de Física de la Terra i Termodinàmica, Universitat de València, E-46100 Burjassot, Spain

[b]Dept. de Física Aplicada, Universitat Politécnica de València, E-46022 València, Spain

[c]Allen Discovery Center, Tufts University, Medford, MA, 02155-4243, USA

Additional experiments concerning the effect of the number of pores, the memristive inductive and capacitative responses of the membrane, and the retention times, together with model theoretical results, are provided.

Effects due to the number of pores

The density of pores on the multipore membranes is an important experimental parameter.[1,2] Here, we have used samples with *ca.* 300 pores/cm^2 and an exposed membrane area of 1 cm^2. The samples show I–V curves with capacitive ($V < 0$) and inductive ($V > 0$) loops (Figure S1). By decreasing the number of pores at constant membrane area the capacitive effects increase, tending to obscure the inductive effects in Figure S1. Then, the conductance potentiation is too low to permit RC computing.

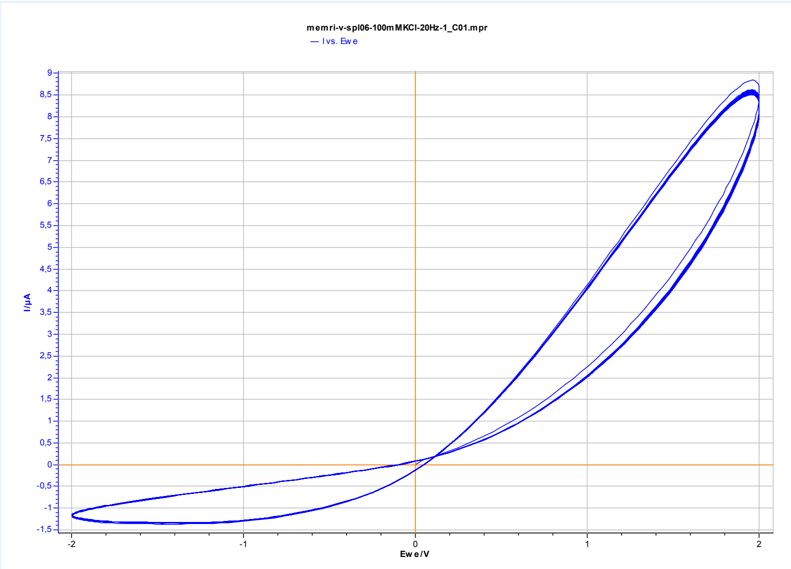

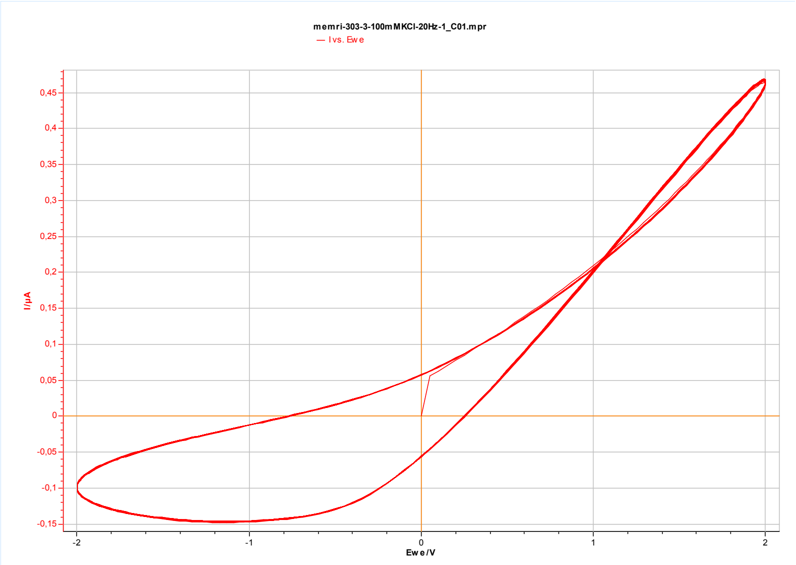

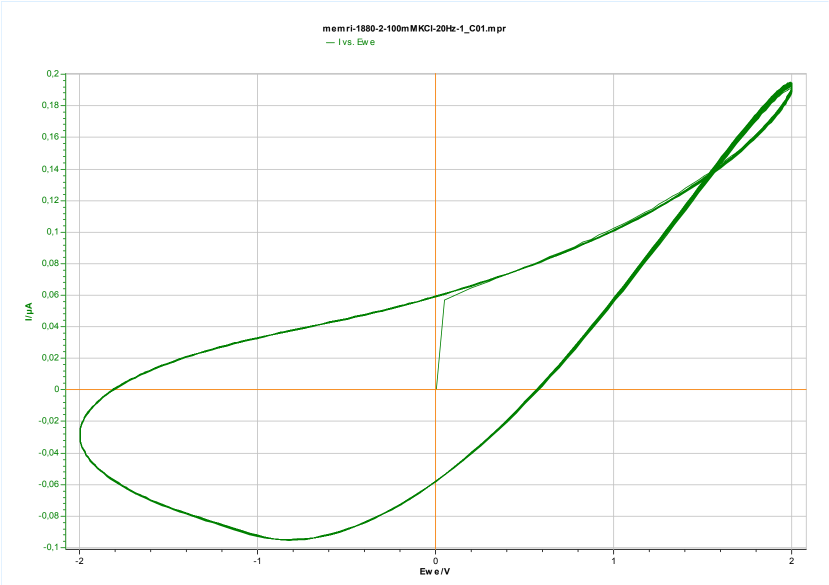

Figure S1. The experimental current (I) – voltage (V) curves of membranes fabricated by irradiation of a polymer foil (polyethylene terephthalate) with *ca*. 300 conical nanopores (*top*), 6 – 7 nanopores (*intermediate*), and a single nanopore (*bottom*).

Memristive inductive and capacitative responses

The antiparallel arrangement of the pores in Figure 5 shows a three-loop I–V curve rather than the usual two-loop curve because two inductive regions with counterclockwise current loops

can now exist at both $V > 0$ and $V < 0$ while a capacitive region with a clockwise current loop is obtained around the origin (Figure S2).[3-6] This memristive effect is reproducible and in agreement with the concavity changes observed in the corresponding steady state I–V curve, as described previously.[4]

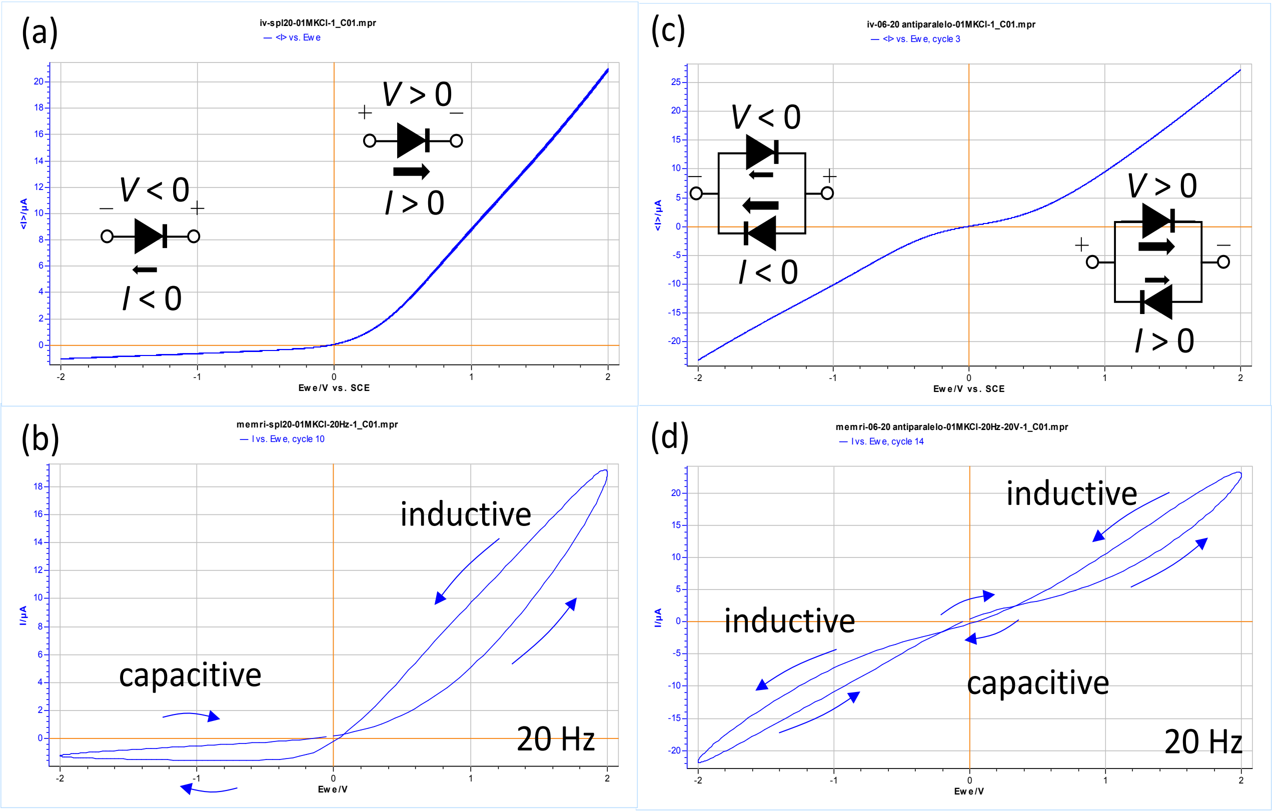

Figure S2. The experimental I–V curves clearly show liquid-state memristive and diode-like effects (*insets*). In the case of a single membrane (a), the steady-state curve displays high ($V > 0$) and low ($V < 0$) conductance states. At high enough driving signal frequencies, inductive ($V > 0$) and capacitive ($V > 0$) loops are apparent (b). However, the robust membrane connectivity also allows antiparallel arrangements showing high conductive branches for both $V > 0$ and $V < 0$ (c). By increasing the signal frequency, the resulting memristive curve presents inductive loops both at $V > 0$ and $V < 0$ that are connected by a small capacitive central loop around $V = 0$ (d). The different experimental choices available[4,5] can increase the RC computing capabilities of the system.

Retention time responses

A set of positive voltage pulses can change the final conductance of the membrane because of the ionic enrichment in the pore. The time duration of the voltage pulse and the time interval between the end of the pulse and the measurement of the current or conductance influence the observed membrane response,[7,8] as shown in the consecutive voltage pulses of Figure S3. The relatively short times involved are usual in nanofluidic systems.

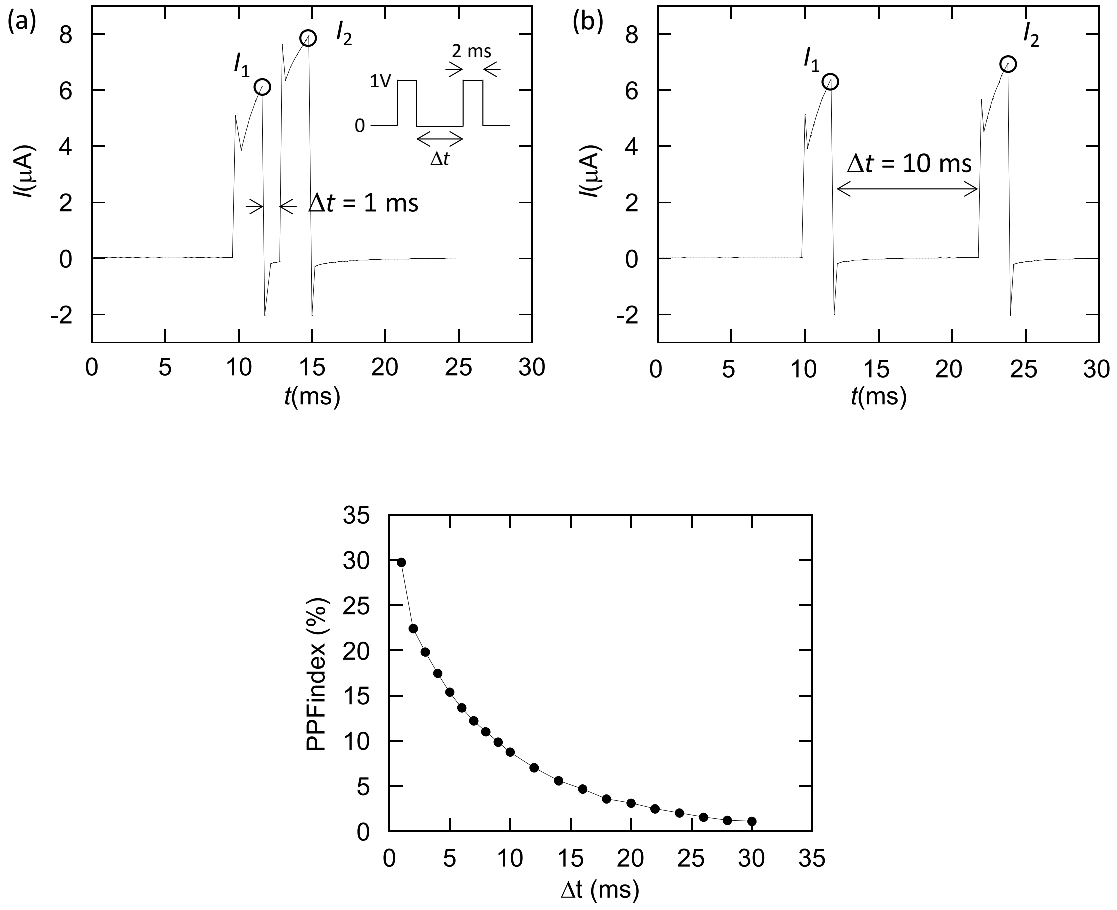

Figure S3. The experimental current-time traces (*top*) obtained with voltage pulses of amplitude 1 V and duration 2 ms, which are separated by the temporal intervals $\Delta t = 1$ ms (a) and 10 ms (b), give the consecutive electrical currents I_1 and I_2, with $I_1 < I_2$. These currents allow to calculate the

Paired Pulse Facilitation index PPF = $100(I_2 - I_1)/I_1$ as a function of Δt (*bottom*), which suggests time responses in the range of ms.

Model theoretical results

We have proposed previously a physical model that accounts for the basic phenomenology observed in memristive asymmetric pores in the case of *slow* driving-voltage signals with characteristic times of the order of 1 s.[5] The fundamental concepts invoked describe the membrane operation on the basis of the electrical interaction between the pore surface charges and the nanoconfined ionic solution. The model has been experimentally validated by studying the nanofluidic tunability as a function of the amplitude and frequency of the driving signal, the ionic concentration, and the solution pH (Figure S4 taken from Reference 5).[5,6,9]

For the sake of completeness, we summarize now how the ionic currents of Figure S4 were obtained from the model equations.[5] First, we assume that the time relaxation of the pore conductance follows that of the counterion concentration (the state variable here) in the pore solution. When a constant electric field $\left(V_0 / L\right)$ is applied across a membrane of thickness L bathed by solutions of ionic concentration c, this relaxation can be approximately described by the continuity equation:

$$\frac{\partial c_i}{\partial t} = -\frac{\partial J_i}{\partial x} \rightarrow \begin{cases} X_+ / \tau_{+0} \approx \dfrac{F}{RT} Dc \dfrac{\left(V_0 / L\right)}{L} \\ X_- / \tau_{-0} \approx \dfrac{F}{RT} Dc \dfrac{\left(V_0 / L\right)}{L} \end{cases} \quad (1)$$

at axial position x, where the times τ_{+0} and τ_{-0} characterize the changes in the ionic concentrations of the pore solution. The current rectification due to the conical pore asymmetry demands to consider separately the cases: (*i*) the cations (majority carriers here) enter the negatively-charged

pore through the tip (positive applied potential, subscript j = +) and (*ii*) the cations enter the pore through the base (negative applied potential, subscript j = -).[5]

The different relaxation times τ_{+0} and τ_{-0} of Equation (1) come from the distinct characteristics of the cone tip and base solutions, where the counterion concentrations are of the order of the respective fixed charge concentrations X_+ (*tip*) and X_- (*base*) because of the local electroneutrality condition. Also, X_+ / X_- should be proportional to the ratio of the pore radii $a_-(base)/a_+(tip) \approx 10-100$ because $X_j = 2|\sigma|/(Fa_j)$, where the surface charge density σ is constant.[9] Thus, the conical pore relaxation can be characterized by two different times corresponding to the tip and base ionic solutions:

$$\tau_{+0} \approx \frac{X_+\left(L^2/D\right)}{cFV_0/(RT)} \qquad \tau_{-0} \approx \frac{X_-\left(L^2/D\right)}{cFV_0/(RT)} \tag{2}$$

The relaxation of the ionic concentrations should then give two distinct equations for the pore conductances $G_j(t)$ (j = +,–) as a function of the external driving signal:[7,13]

$$\frac{\mathrm{d}G_j(t)}{\mathrm{d}t} = -\frac{\left[G_j(t) - G_j^{\mathrm{f}}\right]}{\tau_j}, \ j = +, - \tag{3}$$

In Equation (3), G_j^{f} are the limiting values of the conductances and the frequencies $f_j = 1/\tau_j$ (j = +) characterize the pore response driven by the sinusoidal voltage signals $V(t) > 0$ and $V(t) < 0$ (j = –), respectively. Note that the frequencies f_j of Equation (3) are not equal to the frequencies $f_{j0} = 1/\tau_{j0}$ (Equation (2)) when a time-dependent signal $V(t) = V_0 \sin(2\pi ft)$ of frequency f rather

than a constant voltage V_0 signal (Equation (1)) is applied. In the first case, the external voltage oscillates between V_0 and $-V_0$ and the system response in terms of frequencies f_+ and f_- could depend not only on f_{+0} and f_{-0} but also on the driving frequency f.[5]

For slow enough (*zero* frequency limit) of the external driving signal,[5] we can change V_0 for $V(t)$ in Equation (2) in order to estimate the finite frequencies f_j of Equation (3). By integrating over time the latter equation, we obtain:

$$\begin{aligned} G_+(t) &\approx G_+^{\mathrm{f}} + (G_+^{\mathrm{i}} - G_+^{\mathrm{f}})\exp\left\{-\frac{f_{+0}}{2\pi f}\left[1-\cos(2\pi ft)\right]\right\},\ 0<t<1/2f \\ G_-(t) &\approx G_-^{\mathrm{f}} + (G_-^{\mathrm{i}} - G_-^{\mathrm{f}})\exp\left\{-\frac{f_{-0}}{2\pi f}\left[1+\cos(2\pi ft)\right]\right\},\ 1/2f<t<1/f \end{aligned} \tag{4}$$

In Equation (4), G_j^{i} are the initial values of the conductances corresponding to the sinusoidal signals $V(t) > 0$ ($j = +$) and $V(t) < 0$ ($j = -$), respectively. Note that the conductances $G_j(t)$ can follow the electric potential $V(t)$ changes only after a delay time.[5] From Equation (4), we obtain the currents $I_j(t) = G_j(t)V(t)$ ($j = +,-$) of Figure S4 as:

$$\begin{aligned} I_+(t)/I_0 &= \left\{1+(r-1)\exp\left\{-\frac{f_{+0}}{2\pi f}\left[1-\cos(2\pi ft)\right]\right\}\right\}\sin(2\pi ft),\ 0<t<1/2f,\ V(t)>0 \\ I_-(t)/I_0 &= \left\{r-(r-1)\exp\left\{-\frac{f_{-0}}{2\pi f}\left[1+\cos(2\pi ft)\right]\right\}\right\}\sin(2\pi ft),\ 1/2f<t<1/f,\ V(t)<0 \end{aligned} \tag{5}$$

In Equation (5), $I_0 = G_0V_0$ is a reference current that can be obtained from the reference pore conductance G_0 at the voltage amplitude V_0. Also, the experimental conductance ratio $r = G_+^{\mathrm{i}}/G_+^{\mathrm{f}} = G_-^{\mathrm{f}}/G_-^{\mathrm{i}}$ corresponds to the initial (i) and final (f) limiting conductances at voltages $V > 0$ ($j = +$) and $V < 0$ ($j = -$). A relatively small capacitive current[5] can be added to the conductive

currents of Equation (5) to give the total current. Although this current is much lower than the conductive current,[5] it can be observed experimentally because of the shift in the non-zero crossing point of the I–V curves.[5]

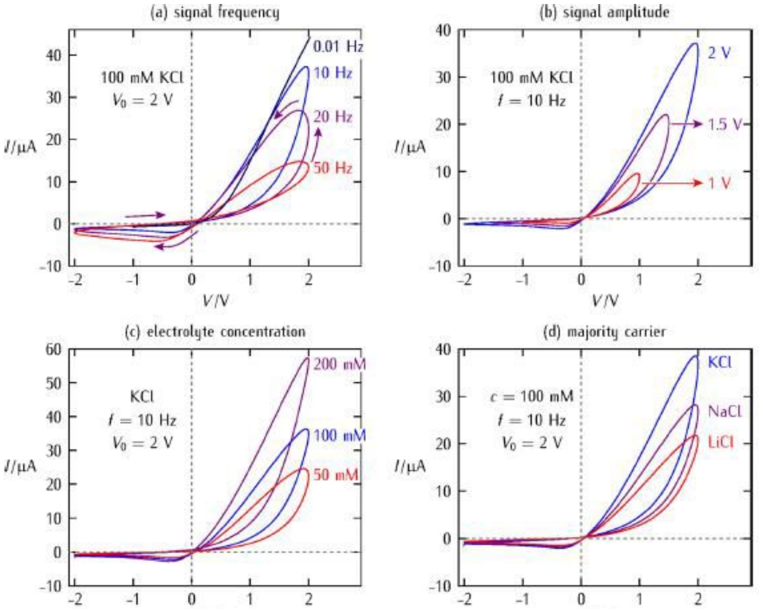

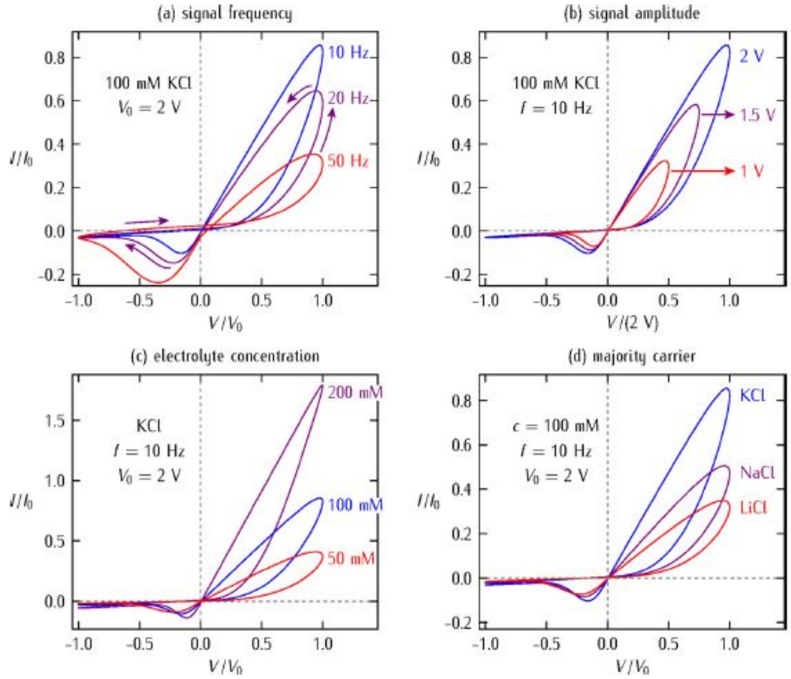

Figure S4. Experimental data (*top*) and model simulation (*bottom*) results for different signal frequencies, voltage amplitudes, ionic concentrations, and salts. Note the ionic conduction and rich

memristive behavior demonstrated experimentally and simulated theoretically. Results taken from Reference 5.

We have also studied theoretically the case of *fast* driving-voltage signals with characteristic times of the order of 1 ms, describing the potentiation and depression of the membrane conductance following different voltage pulses.[10] In this case, the model equations are different from those shown above and can simulate the conical nanopores in the physical reservoir that are used here for digit classification. Note that a set of different voltage pulses can change the final conductance state of the membrane because of the changes in the ionic concentration of the pore solution.[9] The resulting ion accumulation or depletion depends on the positive or negative voltage pulse sign.[10] Also, the time needed for the pore solution to relax to the original state can be modulated by the duration of the pulse and the time established between the end of the pulse and the measurement of the membrane conductance.

In order to implement the digit classification task, we consider a sequence of 4 binary signals V_j (j = 1,…,4) in the form of constant voltage pulses of 2 ms duration, where the bit 1 corresponds to the +4 V pulse and the bit 0 to the −2 V pulse. All bits in the sequence are applied sequentially, without separation between them. Then, the pore conductance $G_j(t)$ corresponding to a particular bit j should follow the voltage changes with a characteristic relaxation time time τ_i as

$$\frac{\mathrm{d}G_j(t)}{\mathrm{d}t} = -\frac{\left[G_j(t) - G_i^{\mathrm{f}}\right]}{\tau_i},\ i = 1,0 \tag{6}$$

where G_i^{f} are the limiting quasi-steady state conductances[5] in the cases of positive (i = 1) and negative (i = 0) voltages, with $\tau_1 = \tau_{+0}$ and $\tau_0 = \tau_{-0}$. By integrating Equation (6) for each bit, we obtain the conductance trace for the 4-bit sequence. The final conductance value characterizes the reservoir state in the digit classification task, as shown in Figure S5 for the binary sequences corresponding to digit “2”. From the conductance, the current can be obtained as $I = GV$ at each voltage V.

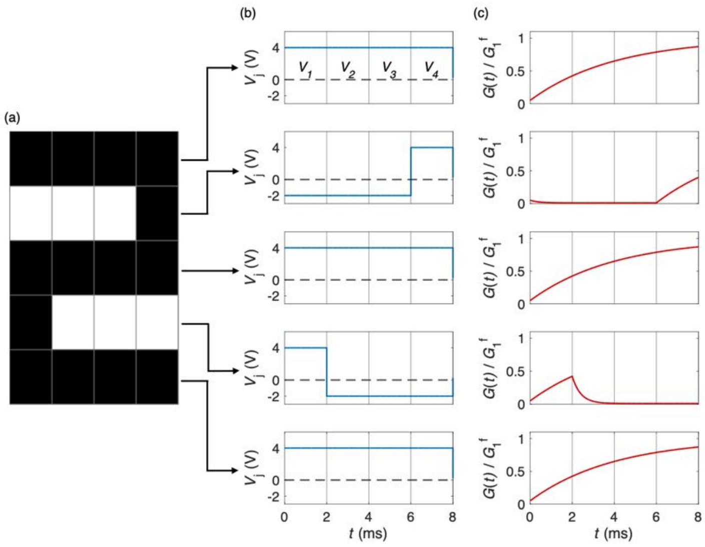

Figure S5. (a) Binary 5 x 4 image of digit “2”. (b) The corresponding voltage sequences. (c) The resulting conductance traces. The conductance is obtained in terms of the quasi-steady value at voltage +4 V, G_1^{f}, and the time parameters τ_{+0} = 4 ms and τ_{-0} = 0.2 ms.

References

(1) Apel, P. Y.; Korchev, Y. E.; Siwy, Z.; Spohr, R.; Yoshida, M. Diode-like single-ion track membrane prepared by electro-stopping. *Nucl. Instrum. Methods Phys. Res., Sect. B* **2001**, *184*, 337–346.

(2) Ma, T.; Janot, J. M.; Balme, S. Track-etched nanopore/membrane: From fundamental to applications. *Small Methods* **2020**, *4*, 2000366.

(3) Bisquert, J. Inductive and capacitive hysteresis of current-voltage curves: Unified structural dynamics in solar energy devices, memristors, ionic transistors, and bioelectronics. *PRX Energy* **2024**, *3*, 011001.

(4) Ramirez, P.; Portillo, S.; Cervera, J.; Bisquert, J.; Mafe, S. Memristive arrangements of nanofluidic pores*, Phys. Rev. E* **2024**, *109*, 044803.

(5) Cervera, J.; Portillo, S.; Ramirez, P.; Mafe, S. Modeling of memory effects in nanofluidic diodes. *Phys. Fluids* **2024**, *36*, 047129.

(6) Ramirez, P.; Gómez, V.; Cervera, J.; Mafe, S.; Bisquert, J. Synaptical tunability of multipore nanofluidic memristors. *J. Phys. Chem. Lett.* **2023**, *14*, 10930.

(7) Kamsma, T. M.; Kim, J.; Kim, K.; Boon, W. Q.; Spitoni, C.; Park, J.; van Roij, R. Brain-inspired computing with fluidic iontronic nanochannels. *Proc. Natl. Acad. Sci. U.S.A.* **2024**, *121*, e2320242121.

(8) Kamsma, T.; Rossing, E.; Spitoni, C.; van Roij, R. Advanced iontronic spiking modes with multiscale diffusive dynamics in a fluidic circuit. *Neuromorphic Comput. Eng.* **2024**, *4*, 024003.

(9) Cervera, J.; Schiedt, B.; Neumann, R.; Mafe, S.; Ramirez, P. Ionic conduction, rectification, and selectivity in single conical nanopores. *J. Chem. Phys.* **2006**, *124*, 104706.

(10) Portillo, S.; Cervera, J.; Mafe, S.; Ramirez, P. Reversible logic with a nanofluidic memristor. *Phys. Rev. E* **2024**, *110*, 065101.
